# The Treatment of Constipation

**Published:** 1909-05-15

**Authors:** Seymour Taylor

**Affiliations:** Physician to the West London Hospital


### Hospital Clinics.

### THE TREATMENT OF CONSTIPATION.

By SEYMOUR TAYLOR, M.D., F.R.C.P.; Physician to the West London Hospital.

(Abstract of a lecture delivered at the West London Post-Graduate College.)

When I started to prepare this lecture I consulted a large number of authorities, but I could learn very little that is new, and it occurred to me that it might be better if I spoke to you of my own experience rather than gave you a *résumé* of other people's work.

To offer, first of all, a definition of constipation, we may say it is a disorder, usually functional, in which a daily relief of the bowels is not naturally obtained. I use the term "functional" so as to exclude gross lesions of the intestine which may be the cause of actual obstruction.

### Ætiological Factors.

As to causation, remember that heredity is a marked feature in some families. Constipation may exist in some members of a family and be absent in others. I know a family of five in which three are habitually constipated, the others not; and another family of two, one of whom has this trouble, the other just the reverse. And yet the environment and food of the members of these families respectively are identical. In many families the various children differ as they favour one or other progenitor who may suffer from this disorder. I know some children in whom the father's or the mother's characteristics are reproduced.

Girls sometimes favour their father in feature, voice, and in other respects, and suffer also from constipation if he does. Boys, again, may resemble their mother in the same tendency, as well as in physical characters. In other families, again, heredity being a well-established fact, all the members may suffer from constipation when both parents are affected.

We must admit, therefore, that in some people constipation is a matter independent of food, drink, habits, and environment. I know of three generations in one family thus affected in which father, son, and grand-daughter have had to take regularly a dinner pill prescribed originally for the former by a celebrated physician; the girl is now about 17 years of age.

### The Penalty of Civilisation.

But we must also admit that in most cases constipation is a penalty of our advanced civilisation. The lower animals in a state of freedom do not suffer from constipation, but when kept in confinement they may do so, and one of the keepers at the Zoo tells me that the lions defæcate on the average but once in four days. Further, amongst nomadic people, gipsies, tramps, showmen, and so on, I find by inquiry that this disorder is comparatively unknown. Those people who enjoy a scanty, but sufficient, dietary suffer less from constipation than do more prosperous folk, who, as a rule, overeat themselves.

As showing the prevalence of constipation, I need only refer to the advertisements seen everywhere in which quack remedies are extolled. I think anyone coming into this country would be justified in concluding that the majority of the inhabitants must be suffering from this condition; and as far as the females are concerned I think he would be right. I estimate that in our urban population 60 per cent. of the women suffer from constipation.

### The Factors of Sex and Modesty.

What are the causes of this? The first is habit—indifference to a daily evacuation. It is wonderful what a little discomfort some people suffer from it. They say they keep in good health, feel well, have no headache nor indigestion, although they frequently go five days without an action of the bowels. Another factor, in women especially, is carelessness. Unless prompted by a desire to evacuate, there is no self-inquiry as to whether it is necessary to have an evacuation or not. That is so in women, whereas their husbands or brothers are not careless and indifferent in this way. The latter do not think of starting off for business until they have emptied the bowel, because they know that they will suffer if they do. Another cause is a sense of false modesty or shyness in being seen going to the closet. I do not want to appear gross, but this is a very important factor whch you must recognise, and of which you should warn the mother of a family of growing daughters. Such shyness does not obtain on the Continent. I am not sure that I do not admire the shy English girl rather than her sisters on the other side of the Channel; but be that as it may, this shyness is one of the causes of habitual constipation. I have had to speak of this to ladies who have told me that they do not like to be seen going into a closet even by a housemaid who may be dusting the stairs. They will, even when they have a closet on their own landing close to their bedroom, postpone the act because someone (of their own sex) is about. Such procrastination is fatal; the moisture of the fæces is reabsorbed into the system, and a hard mass is formed and retained.

### Diet and Constipation.

The next heading of the subject is diet. The man or woman whose food is too much in quantity or too rich in quality gets an over-distended bowel which becomes sluggish. The rich man who eats a superabundance of food suffers from constipation. The woman who marries a rich man may hitherto have lived sparingly; but she gets into the habit of eating a big lunch and a rich tea of buttered toast and cakes, and then gets a fictitious appetite for an indigestible dinner by driving round the Park.

Another cause of constipation is an excess of butchers' meat, which is rich in nitrogenous products and poor in refuse. Much of it is absorbed, and but little remains to stimulate peristalsis. On the other hand, vegetables and vegetable foods may be too scanty in our allowance, with a like result. Another article of diet which must be mentioned is milk, particularly in children. Small quantities of milk sipped or cooked in milk puddings do no harm; but the anxious mother who allows her child to go to the larder the moment he comes in and gulp down milk is promoting constipation in him. Then, too, food which is too well ground or too well prepared has the same tendency. Our bread, ground between steel rollers; our beefsteak, free from all gristle; the leg of mutton with the outside carefully cut off and left at the edge of the plate—all these tend to produce constipation, because these pieces of gristle and skin, though low in nutritive power, stimulate the bowel.

### Direct Causes.

Those, gentlemen, are the predisposing causes of the costive habit. The direct causes come under the heads of secretion and the muscular apparatus. If you remember your physiology, bile is Nature's purge, and therefore those who have an inactive liver will suffer from constipation, and the stool in this case is pale in colour. In those who eat too small an amount of food, whether from choice or necessity, the bowel is not stimulated as it should be by the passage of food down its interior; muscular inactivity is thus a factor in such circumstances.

Suppose the secretions are diminished. This may be brought about by the absence of certain foods which ought normally to set up the production of these secretions; and the formation of gas in the intestines is an essential part of the cure of such cases. One of the reasons that vegetable food produces a lax condition of the bowel is that by decomposition it gives rise to certain gases which stimulate the intestine, not only to peristalsis, but also to increased secretion.

It is a well-known fact that fermented liquors such as beer and champagne will undoubtedly act as aperients to certain people. I know a man, the subject of the costive habit, who will not take aperients; when he gets constipated he goes to a certain restaurant and orders a simple meal and a pint of stout with it. After this he has to hurry home at once, because he knows that in an hour's time he will have an action as if he had taken a brisk purge. So much is this property of beer the fact that at Burton-on-Trent there is a popular saying, "Did you ever see a costive brewer?" This has passed into a proverb, because all the workmen from the highest to the lowest are allowed free a certain amount of beer a day.

### The Factors of Insufficient Fluid.

The secretions, again, of the intestine may be insufficient because there is not enough food taken to stimulate the lower intestine—a point not recognised as it should be. Looped up to the stomach, but 18 feet or more away, is the transverse colon; and one of the reasons that a man has a desire to evacuate the bowels just after breakfast is that the food in the stomach stimulates the transverse colon so that evacuation results.

So it comes about that a man who is not well fed, and especially if he does not eat a proper breakfast, may gradually acquire the costive habit. I have often thought about the reason why the transverse colon is looped to the stomach. One eats a bit of indigestible food, such as a piece of half-chewed apple; in less than a quarter of an hour there is intense colic, which is not relieved until the bowel is emptied both of fæces and flatulence. There is, in fact, a close reflex connection between the stomach and the transverse colon, for they have to a large extent the same blood supply and the same nervous supply. The effect of this connection is seen by the draught of warm water or tea taken in the early morning, which is very useful in preventing constipation. No one will persuade me that this fluid can possibly traverse, in three-quarters of an hour or so, the entire alimentary canal; the effect must be largely a reflex.

Constipation may be due to wasting of the involuntary muscles, as in fevers; or to wasting of the abdominal muscles, as in pendulous belly; or wasting may be brought on by the very disease which it has caused. The belly muscles suffer from inertia, and the man who takes no exercise suffers, of course, from constipation. You see that, too, in the lower animals; as soon as your dog is let out of doors he goes for a scamper, and in ten minutes the bowels are relieved.

Want of nerve energy is another cause of constipation. The indolent creature who loves his armchair suffers from want of nerve energy and constipation. On the other hand, the energetic man who rushes off for his train may lose the opportunity and not get his relief until the next day.

### Rational Treatment.

I must say a few words now as to the treatment of this disease, which in functional cases is perhaps not so much a disease as a disorder. Anyone can write a prescription for a purge; any chemist can do it excellently, and the daily Press is full of advertisements of patent remedies. But the best treatment is that which will overcome the trouble without the use of drugs.

Let the patient select some hour of the day, either morning or evening, when he can always have the opportunity to attempt to coax nature. If after breakfast is not convenient, the middle of the morning, or last thing before bed time will do as well. Tell him to go regularly every day at the same time. He must not sit and strain; but if after 10 minutes no action results let him give up the attempt and renew it next day at the same hour. After a few days it will be found that the bowel will get accustomed to keeping this appointment.

When building a house the closets should be made with a low seat. I do not want to go into offensive details, but it must be remembered that savage man in the natural attitude of defæcation squats with his chin on his knees and his abdomen pressed on by his thighs. Therefore the seat should shelve backwards, and you will be astonished not only at the ease with which evacuation is thus obtained, but also by the freedom and amount of it.

### Drugs in Chronic Constipation.

I think it is a matter of little moment what drug you select; aloes is a constituent of most purges. One of the most successful patent medicines on the market is one in which the purgative principle is present in very small quantity; thus the patient can take one or two or three or more pills according to his experience of what he requires. Whatever you give, let it be in small continued doses. If you like, order $\frac{1}{10}$ grain of calomel every night, followed by a saline aperient in the morning. Another favourite prescription of mine is $\frac{1}{8}$ grain of podophyllin every night for ten or twelve nights, combined always with a regular visit to the closet at a fixed time. Iridin and enonymin are also valuable, separately or combined; if you give these with podophyllin, $\frac{1}{16}$ grain of each will be enough.

Great advantage will accrue to the sufferer from constipation if you follow up this by advising one or two apples in the early morning; the malic acid in them helps considerably the effect of the drugs. If the patient objects to apples, you may supplement the effect by one of the saline aperient waters. But I caution you that if you prescribe these by themselves you will have disappointments. They create noise and rumbling and borborygmi, but produce little or no relief. Strychnine has been extolled as an adjuvant to these purgatives, but it is in my experience only useful in young people. There comes a time of life, say after the fiftieth year, when strychnine is of little or no use for the purpose of emptying the intestines.

### Other Lines of Treatment.

You must enjoin also as much exercise as the patient can obtain, or, failing that, abdominal massage. You are perhaps not aware of the fact that it is possible to knead the gall bladder through the abdominal wall by means of massage. I have once or twice actually set up biliary colic as the result of thorough examination of the abdomen for suspected gall-stones.

Another way of overcoming the difficulty is by the use of enemata. Do not be frightened of enemata; I see no danger in them at all, and the practice is a common one on the Continent. One of the best ways is to give three or four ounces of olive oil, and a common soap enema an hour afterwards. On the next day the quantity of oil may be reduced to one ounce; and the patient ought also to be instructed to wear a pilch or napkin to prevent the oil exuding through the sphincter.

There is also something that must be said about surgical treatment. Search the anus for fissure, one of the most fertile causes of constipation in elderly women. This can in many cases be cured by getting the patient to wash the anus after each evacuation and to wear a small piece of boric wool over the part. If piles are present they should be removed or otherwise treated.

But when I read that the patient's large intestine should be taken away *en bloc*, or that, through a hole in the abdominal wall, medicaments should be injected, all I can say is that you will not get your patients to consent to such measures. We must not let surgeons go to such an extreme as to advocate intestinal anastomosis or the removal of six feet of the intestine for a condition which should be, and is, amenable to medical treatment. Speaking seriously, I should never advise any patient to undergo these severe operations; and I repeat that we ought to be able to cure this disease of chronic constipation by the means which I have laid down in this lecture.

